# 914 Daily Rounds and Length of Stay: Housed versus Unhoused

**DOI:** 10.1093/jbcr/iraf019.445

**Published:** 2025-04-01

**Authors:** Paul Kantor

**Affiliations:** Torrance Memorial Medical Center

## Abstract

**Introduction:**

Burn patients’ length of stay (LOS) are complicated by the extent of injury and inpatient interventions. Complexities (not limited to) wound care and surgical needs can delay discharge; housing situations impose added factors for hospital systems to account for when planning exit strategies. Charges for an overnight stay in the intensive care unit (ICU) vary based on several factors and acuity of injury; In 2021, average overnight costs in Burn Unit Intensive Care (BICU) was $4,181, but costs vary based on burn injury acuity (KFF.org).

**Methods:**

An analysis utilizing the national burn database to gather information about discharged patients from TMMC BICU between June 2022 and June 2024. Average LOS was calculated and separated by the patient’s documented housing situation. The “LOS Without Surgery” was targeted to show differences in discharge timelines for patients with less complicated exit strategies and home care needs. LOS with Surgical Intervention shows a mixed acuity patient population with at least one OR day. An Excel spreadsheet was utilized to calculate the average LOS in each category and the total sample size. LOS housed population n = 303; unhoused n = 35. It should be noted that the unhoused population sample size was significantly smaller following rounds implementation with n = 12.

**Results:**

An initial analysis of Burn Registry data showed unhoused individuals’ LOS was 2.75 and 1.1 days longer than housed individuals with and without surgical intervention respectively. Following daily rounds of implementation with CM and LCSW, housed individuals without surgical intervention saw a decrease in LOS by 0.3 hospital days; unhoused individuals saw an increase in LOS by 1.3 days. Similarly, following implementing daily rounds with CM and LCSW, housed individuals with surgical intervention saw a decrease in LOS by 3.1 days; unhoused individuals saw an increase of 25.8 days.

**Conclusions:**

Case management (CM) and social workers (LCSW) work directly with patients, RNs, MDs, and community partners to plan discharges. Communication between nursing and ancillary varies between institutions, however. Levin et al. (2020) notes that regular rounding focused on discharge planning has been proven to prevent delays in discharge. Implementing discharge planning rounds not only promotes education for nurses but also develops stronger relationships between stakeholders and patients. While the sample size in the unhoused population limits

**Applicability of Research to Practice:**

Burn victims leave the hospital setting healing, not fully healed, creating significant opportunities in the outpatient setting. Specialized care opportunities come in many forms depending on the patients’ needs whether it be care being delivered at home or a specialized facility, discharge planning from the inpatient setting should be initiated at the first opportunity

**Funding for the Study:**

N/A

